# Single-Incision Laparoscopy in Abdominal Trauma: Current Evidence, Clinical Applications, and Evolving Role—A Narrative Review

**DOI:** 10.3390/jcm14103610

**Published:** 2025-05-21

**Authors:** Sebeom Jeon, Kang-Kook Choi

**Affiliations:** 1 Department of Trauma Surgery, Gachon University Gil Medical Center, Incheon 21565, Republic of Korea; dsjeonse@gmail.com; 2 Department of Traumatology, Gachon University College of Medicine, Incheon 21565, Republic of Korea

**Keywords:** laparoscopy, trauma, clinical application, single-incision laparoscopy

## Abstract

**Background/Objectives:** Laparoscopic surgery plays a central role in the management of abdominal trauma, particularly in patients with hemodynamic stability. Recently, single-port laparoscopic surgery (SPLS) has emerged as a technique that further reduces access-related trauma while preserving the benefits of conventional laparoscopy. Thus, this review aimed to examine the current landscape of SPLS in trauma care, summarizing available clinical data and highlighting practical considerations for its use. Despite the limited experience, early evidence suggests that SPLS can achieve diagnostic and therapeutic outcomes comparable to those achieved by multi-port approaches in selected cases. Particular attention is given to a hybrid method that combines intracorporeal assessment with extracorporeal small bowel examination and repair through a single umbilical incision. This technique offers a pragmatic balance between thorough exploration and minimal invasiveness. **Methods**: We searched PubMed, Scopus, Web of Science, and Google Scholar through December 2023 using the terms “single-port laparoscopy”, “single-incision laparoscopic surgery”, “trauma laparoscopy”, and related phrases. Case reports, case series, comparative studies, and reviews describing single-incision laparoscopic surgery in trauma were included in this narrative analysis. **Results**: SPLS may offer advantages in terms of postoperative pain, wound complications, and cosmetic outcomes, especially in younger patients. As familiarity with the approach increases and dedicated instrumentation becomes more accessible, its role in trauma protocols may expand. **Conclusions**: Further prospective research is needed to define long-term outcomes, refine patient selection, and integrate SPLS more systematically into trauma care protocols.

## 1. Introduction

Laparoscopic surgery is an invaluable tool for managing abdominal trauma. Since its first reported use in patients with trauma in the 1970s [1,2], the role of laparoscopy has expanded from purely diagnostic purposes to therapeutic interventions. Compared to open laparotomy, laparoscopy offers numerous advantages, such as reduced postoperative pain, lower wound complication rates, shorter hospital stays, and faster recovery [3,4]. Importantly, diagnostic laparoscopy can accurately identify intra-abdominal injuries with high sensitivity, helping avoid unnecessary non-therapeutic (negative) laparotomies that carry significant risk of morbidity [5,6]. In fact, the selective use of diagnostic laparoscopy in trauma can prevent a negative laparotomy in roughly 20–50% of cases [7,8], sparing patients the pain and complications of an unnecessary open surgery. Over the past few decades, the emergence of novel, minimally invasive techniques has further advanced the boundaries of trauma care. Notably, advances like natural orifice transluminal endoscopic surgery (NOTES) and single-port laparoscopic surgery (SPLS) have been developed in an effort to achieve “scarless” surgery and minimize abdominal wall trauma [9]. Although the NOTES approach was heavily explored, it has faced technical challenges—requiring specialized instruments, steep learning curves, and prolonged operative times—which have limited its adoption [9,10]. In contrast, single-port laparoscopy has evolved rapidly as a feasible alternative in many surgical fields. SPLS, also known as single-incision laparoscopic surgery (SILS) or single-port access surgery, involves laparoscopy through a single incision (usually at the umbilicus) instead of multiple ports. This approach aims to further reduce the invasiveness of surgery by eliminating additional trocar sites, thereby potentially improving cosmetic outcomes and reducing incisional pain and morbidity [11,12]. In trauma surgery, where patients are often young and the avoidance of multiple scars is desirable, SPLS represents the modern evolution of minimally invasive techniques. It maintains all the proven benefits of standard laparoscopy while attempting to minimize the access trauma even further [13,14]. However, the role of SPLS in abdominal trauma management remains unclear. This narrative review discusses the current status of laparoscopic surgery in trauma, outlines the evolution of single-port techniques, examines the existing evidence for SPLS in trauma (including case series and the single comparative study), and considers technical aspects, patient selection, and future directions. This review emphasizes that SPLS is a contemporary alternative to traditional multi-port laparoscopy for the care of patients with abdominal trauma.

## 2. Methods

We performed a comprehensive literature search to gather relevant articles on single-incision laparoscopic surgery in trauma. Databases such as PubMed, Google Scholar, Scopus, and Web of Science were used, and we searched for publications up to December 2023. Key search terms included “single-port laparoscopy”, “single-incision laparoscopic surgery”, “trauma laparoscopy”, and related phrases. We included case reports, case series, comparative studies, and review articles in our narrative analysis, without language restrictions. As this is a narrative review (not a systematic review), our goal was to capture a broad range of published experiences with SPLS in trauma rather than to perform a formal meta-analysis.

## 3. Conventional Laparoscopy in Abdominal Trauma

### 3.1. Techniques

Laparoscopy plays diagnostic and therapeutic roles in trauma care. Diagnostic laparoscopy allows direct visualization of the abdominal cavity to identify injuries to the diaphragm, solid organs, or bowels and can guide the need for further intervention without a full exploratory laparotomy. Therapeutic laparoscopy involves performing necessary repairs or interventions laparoscopically, such as achieving hemostasis, suturing a perforation, or resecting a damaged organ, thereby treating the injury in a minimally invasive manner. Since the 1990s, reports of therapeutic laparoscopy in trauma have demonstrated that many injuries can be repaired laparoscopically in select patients [15,16,17]. Today, many trauma centers incorporate laparoscopy as part of their algorithm for patients with hemodynamic stability with suspected intra-abdominal injuries. For example, a common multi-port setup uses an infra-umbilical 10 mm port for the laparoscope and two or more additional 5 mm working ports in the right and left upper quadrants or iliac fossae [13]. Using the camera port, the surgeon systematically inspected the abdomen for blood, bile, intestinal contents, and organ injury. The liver and spleen are examined for lacerations; the entire bowel is “run” from the stomach and duodenum down to the rectum, and the mesentery, bladder, and both hemidiaphragms are carefully inspected [13]. Atraumatic graspers inserted through the lateral ports are used to elevate and move bowel loops, often employing a cross-handed technique to improve the exposure of both sides of the intestine [13]. This methodological exploration ensured that even subtle injuries (e.g., small serosal tears or mesenteric bleeders) could be detected and addressed. If an injury is identified, a range of minimally invasive techniques can be employed. Solid organ bleeding can be controlled with cautery or topical hemostatic agents, whereas bowel perforations can be closed with intracorporeal suturing or stapling. In some cases, a laparoscopy-assisted approach is used, such as exteriorizing a bowel segment through a slightly enlarged port site for repair or resection (mini-laparotomy), which maintains minimal invasiveness while facilitating complex repairs [13]. Throughout any trauma laparoscopy, the surgical team remains prepared to convert to an open laparotomy if needed for patient safety or if the laparoscopic examination is unsatisfactory [18,19].

### 3.2. Outcomes and Benefits

Conventional laparoscopy for trauma shows excellent results when applied to appropriate patients. In experienced hands and with proper patient selection (typically patients with hemodynamic stability without obvious need for immediate open surgery), laparoscopy can reduce the rate of unnecessary laparotomies and associated morbidity [3,8,20,21]. For example, one series reported that implementing diagnostic laparoscopy reduced non-therapeutic laparotomies by approximately 19%, with no missed injuries in the laparoscopy group [8]. Multiple studies and meta-analyses have confirmed that in patients with stable trauma, laparoscopic management is safe and yields outcomes at least equivalent to those of laparotomy. A 2021 systematic review of laparoscopy for blunt abdominal trauma revealed that laparoscopy significantly decreased postoperative pain and hospital length of stay, with no increase in missed injuries or mortality, compared to open surgery [3]. Similarly, another meta-analysis in 2022, including both blunt and penetrating trauma, reported no significant difference in the missed injury rate or overall mortality between patients managed laparoscopically and those undergoing immediate laparotomy [4]. In addition, patients managed with laparoscopy tend to experience fewer wound-related complications (such as infections or hernias) and shorter hospitalizations than those who underwent open surgery [4,14]. In a single-center 7-year review, Lim et al. noted significantly lower wound infection rates and shorter recovery times in the laparoscopy group, with similar operative durations and zero missed intra-abdominal injuries or abscesses, when compared with laparotomy [14]. Notably, the conversion rate to open surgery in that study was 18%, indicating that a substantial subset of patients could be definitively managed without conversion [14]. Other centers have reported conversion rates in the range of 25–30% when applying laparoscopy to trauma [8,19], which underscores that while many cases can be completed minimally invasively, the surgeon must be ready to convert in about one out of every 3–4 patients based on findings or limitations of the laparoscopic approach.

### 3.3. Limitations

Despite these advantages, conventional multi-port laparoscopy for trauma has several limitations. The approach is generally limited to patients with hemodynamic stability (those who can tolerate insufflation) and the time required for a thorough laparoscopic examination. Patients with massive hemorrhage or instability usually require immediate laparotomy (open surgery) for the rapid control of bleeding (a situation in which laparoscopy is contraindicated). Even in stable patients, laparoscopy must be meticulously performed to avoid missing injuries. The most common complication of diagnostic laparoscopy is a missed injury due to limited exposure or access [22]. Historical studies reported missed injury rates of nearly 20–40% during the early experience with trauma laparoscopy [10,22], which initially tempered enthusiasm for its use. However, techniques have improved, and adjuncts such as patient repositioning (Trendelenburg or reverse Trendelenburg to visualize different compartments) and hand-assisted methods have helped minimize missed injuries [19]. Indeed, a recent series using meticulous techniques has documented missed injury rates near zero [19]. Other limitations of standard laparoscopy include the inability to easily palpate organs (losing tactile feedback) and technical difficulty in repairing certain injuries laparoscopically (e.g., complex hollow organ injuries or retroperitoneal vascular injuries). Adequate exposure can be challenging for posterior injuries (such as those at the back of the diaphragm or retroperitoneum), sometimes necessitating creative strategies or conversion to open surgery to safely address them [22]. Additionally, multi-port laparoscopic procedures involve multiple small incisions, each of which carries a risk of bleeding, infection, or herniation. Although these risks were lower than those associated with open surgery, they did not reach zero. In summary, conventional trauma laparoscopy requires careful patient selection, advanced surgical skills, and readiness to convert to open surgery if the situation dictates. When these criteria are met, conventional trauma laparoscopy becomes a highly effective tool to reduce morbidity by avoiding many non-therapeutic laparotomies [23].

## 4. Evolution of Single-Port Laparoscopic Techniques

The necessity to further minimize surgical invasiveness has led to the development of single-port laparoscopic techniques. Using one instead of several incisions, surgeons aim to reduce the surgical footprint and perform virtually scarless operations. SPLS was initially described in the early 1990s, first in gynecologic surgery in 1992 and shortly thereafter in general surgery, where the first single-port cholecystectomy was reported in 1997 [11,12]. Early pioneering cases demonstrated the feasibility of performing standard laparoscopic procedures through a single umbilical incision, but enthusiasm was limited in the 1990s due to the lack of specialized instruments and technical difficulty of operating with coaxial instruments [10,13,24]. However, by the mid-2000s, the resurgence of interest in single-incision surgery was fueled by technological improvements. Surgeons in urology and general surgery began exploring single-incision approaches for appendectomies, cholecystectomies, colorectal resections, and other procedures between 2007 and 2008, aided by newly developed equipment [25,26]. The introduction of flexible-tip endoscopes, articulating instruments, and dedicated single-port access devices (such as multi-lumen port systems) has addressed many technical challenges that hampered earlier efforts [13]. Device manufacturers developed commercially available single-port platforms—e.g., the SILS™ Port (Covidien, Dublin, Ireland), TriPort^®^ (trademark of Advanced Surgical Concepts, Wicklow, Ireland), GelPoint^®^ (Applied Medical, Rancho Santa Margarita, CA, USA) and others—which allowed multiple instruments to be inserted through one incision with improved maneuverability [13]. In parallel, surgeons also innovated low-cost solutions, like the “glove port” (using a surgical glove attached to a wound retractor as an improvised multi-instrument port), broadening access to the single-port technique, even in resource-limited settings.

By the 2010s, SPLS had been performed in almost every domain of abdominal surgery. Case series and reports documented single-port appendectomies, cholecystectomies, colorectal resections, splenectomies, adrenalectomies, nephrectomies, gynecologic procedures, and even bariatric surgeries performed entirely through one incision [13,27]. These early experiences suggest that SPLS is generally safe and feasible for experienced surgeons, with outcomes comparable to those of standard multi-port laparoscopy [27]. Patients uniformly appreciated the improved cosmesis of a hidden umbilical scar and potentially fewer incisional sites of pain [10,28]. Nonetheless, the widespread adoption of SPLS in routine practice requires caution. One reason is that high-level evidence (large comparative trials) has been lacking—the literature has consisted mostly of small series and observational studies, with few randomized studies to conclusively demonstrate superiority or non-inferiority [10,27]. In the early 2010s, there was a debate in the surgical community about whether SPLS represented a true advance or simply a cosmetic improvement with a steeper learning curve: “a new toy or a new standard?” [10]. However, confidence in single-port techniques has increased over time as more surgeons have mastered this approach. By eliminating additional trocars, SPLS reduces potential port-site complications and may shorten recovery while maintaining all the benefits of minimally invasive surgery [13,14]. Importantly, if a single-port approach proves insufficient during a case, the surgeon can add additional ports, essentially converting to conventional laparoscopy without the need to revert to a fully open operation [29]. This flexibility ensures that patient safety is not compromised when starting with a single-port attempt.

In the context of trauma surgery, single-port laparoscopy is a natural extension of the trend toward less invasive interventions. Trauma surgeons have observed the developments in elective surgery and have begun to consider whether similar techniques can be employed for patients with trauma. By the late 2010s, a few reports described SPLS use in trauma, although the experience remained limited compared with that in other fields [30]. Notably, a 2017 comparative study by İlhan et al. evaluated a single-incision diagnostic laparoscopy approach in penetrating trauma, providing some of the first evidence in this field (discussed below) [29]. The potential appeal of SPLS in trauma includes cosmetic benefits and practical considerations. With a single well-placed port (usually at the umbilicus), a skilled surgeon may be able to inspect the entire abdomen by angling instruments and the laparoscope, possibly mitigating the issue of suboptimal port placement in an uncertain injury pattern [29]. In multi-port laparoscopy, if the initial ports are placed in locations that are not ideal for the injuries found, additional ports or repositioning might be needed, whereas a single central access point could theoretically allow viewing of all quadrants. This hypothetical advantage remains to be fully proven; however, it is an intriguing concept for trauma, in which the injury location is often unknown at the outset. In summary, SPLS has evolved from an experimental approach into a versatile surgical alternative across several disciplines. The following sections examine how this technique has been applied to trauma cases and the achieved results.

## 5. Evidence for SPLS in Abdominal Trauma

The application of SPLS for trauma is relatively novel, and the evidence remains limited. Most published reports consist of individual case reports or small series. To date, only one comparative study has directly evaluated single-port versus multi-port laparoscopy in patients with trauma. Nevertheless, the available data suggest that SPLS is feasible in selected patients with trauma and can yield diagnostic and therapeutic results similar to those of conventional laparoscopy.

### 5.1. SPLS in Penetrating Abdominal Trauma

The most substantial study of SPLS in trauma was performed by İlhan et al. [29], who reported in 2017 a comparative series of patients with penetrating thoracoabdominal stab wounds managed by either single-incision diagnostic laparoscopy or traditional multi-incision diagnostic laparoscopy [29]. This retrospective study included 102 patients with hemodynamic stability with left anterior thoracoabdominal stab injuries. Twenty-six patients underwent single-incision laparoscopy using a trans-umbilical port, and seventy-six patients underwent standard multi-port laparoscopy [29]. The primary focus was the evaluation of the left diaphragm for injury because diaphragmatic lacerations are a common concern in such wounds. The findings demonstrated that single-port laparoscopy was equivalent to multi-port laparoscopy. Diaphragmatic injuries were identified in 34.6% and 26.3% of the single-incision diagnostic laparoscopy and multi-incision diagnostic laparoscopy groups, respectively, which was not significantly different [29]. Importantly, no significant differences were observed in the operative time or postoperative complications between the single-port and multi-port groups [29]. None of the patients in either group had a missed diaphragmatic injury at follow-up, indicating that diagnostic efficacy was comparable. The authors concluded that single-incision laparoscopy is a safe and feasible alternative to conventional laparoscopy for penetrating injuries and provides a similar diagnostic yield and therapeutic capability [29]. They suggested that, in experienced hands, a single-port approach could be used to evaluate and even repair diaphragmatic wounds, potentially offering the cosmetic and reduced morbidity benefits of a single incision without compromising patient outcomes [29]. This study is the first to provide comparative evidence supporting SPLS in patients with trauma. Its limitations include the fact that it was non-randomized and focused on a specific injury pattern (left-sided stab wounds). Nevertheless, it provides a proof of concept that single-port techniques can match the performance of standard laparoscopy in trauma scenarios.

Beyond this comparative study, most of the literature reporting SPLS in trauma consists of case reports and small case series. These reports, while anecdotal, illustrate the breadth of situations in which single-port laparoscopy has been attempted. To date, no randomized controlled trials or meta-analyses have been performed specifically on SPLS in trauma, which means that the evidence base for this technique is derived entirely from lower-level studies.

### 5.2. SPLS in Blunt Abdominal Trauma (Small Bowel Injury)

Yang and Kim [31] described a case of blunt abdominal trauma involving small bowel perforation that was managed with single-incision diagnostic laparoscopy [31]. The patient had equivocal computed tomography (CT) findings but was clinically suspected of intestinal injury. A single umbilical port was placed, allowing surgeons to explore the abdomen and identify small perforations in the jejunum. Rather than adding more ports, they enlarged the umbilical incision by a few centimeters (mini-laparotomy), exteriorized the injured bowel loop for repair, and closed the incision; this approach avoids full midline laparotomy. The authors reported that single-port exploration followed by extracorporeal repair was successful. The patient recovered without complications, and they highlighted it as a feasible and safe alternative to conventional multi-port laparoscopy in select blunt trauma cases [31]. This case exemplifies how SPLS can be combined with limited open techniques to achieve the necessary repair while minimizing overall trauma to the abdominal wall.

### 5.3. SPLS in Solid Organ Injuries

Reports on single-port laparoscopic evaluation of solid organ injuries (e.g., spleen or liver lacerations) in trauma are limited and not well documented in the literature. In general, solid organ injuries that require surgical intervention (e.g., high-grade splenic laceration with ongoing bleeding) often require rapid action, and most surgeons would use multiple ports or an open approach for definitive hemostasis [32,33]. However, one can envision performing single-port laparoscopy in patients with stable trauma with suspected isolated solid organ injuries, mainly for diagnostic confirmation. If active bleeding is observed, additional ports can be inserted for the application of clips, coagulation devices, or sutures. No large series specifically addressing single-port laparoscopic splenectomy or hepatorrhaphy in trauma was retrieved, suggesting that this application remains rare.

### 5.4. SPLS in Pediatric Abdominal Trauma

Children might seem ideal candidates for single-incision surgery because of their smaller body size and desire to avoid scarring; however, the literature on pediatric trauma SPLS is virtually nonexistent to date. Anecdotally, some pediatric surgeons have used single-port laparoscopy for appendectomies and other emergent procedures in children [28], which indicates the safety of the technique in younger patients [34]. The extent of trauma likely depends on the surgeon’s experience and comfort. As with adults, careful case selection is key. In studies of pediatric trauma patients that concentrate on conventional multi-port laparoscopy rather than single-incision techniques, laparoscopy is regarded as a feasible and effective approach for both diagnostic and therapeutic purposes [35]. Its use has been linked to fewer negative laparotomies and a reduced incidence of surgical-site infections, highlighting its practical and potential benefits in the management of pediatric abdominal trauma [36]. Nonetheless, adequate hemodynamic stability is a prerequisite, and the procedure must be performed by surgeons with substantial experience and technical proficiency.

Overall, the existing limited evidence indicates that single-port laparoscopy can be used in patients with trauma without loss of diagnostic accuracy or efficacy, at least in certain scenarios. The lack of multiple large studies indicates that the conclusions are tentative. Single-port approaches to trauma have primarily been used for diagnostic exploration (identifying injuries) and relatively straightforward therapeutic maneuvers (simple suture repairs or cauterization). More complex trauma procedures, such as resection and anastomosis of the bowel and splenectomy, have rarely been attempted through a single port alone, likely because the technical difficulty increases with task complexity. In practice, a surgeon may start with a single-port exploration, and if a complex injury is uncovered, ports are added (converting to standard laparoscopy) or open surgery is performed. This staged approach allows one to gain the benefits of SPLS when possible while incurring no penalty if a different approach is necessary [19]. To date, no studies have reported adverse outcomes that are uniquely attributable to the single-port method in trauma, and the published cases report missed injury rates or complications comparable to those of historical series using multi-port laparoscopy [18,19]. This suggests that SPLS is a viable alternative technique for trauma laparoscopy when performed by skilled surgeons, which warrants further investigation.

## 6. Technical Considerations and Patient Selection for SPLS in Abdominal Trauma

Implementing single-port laparoscopy in patients with trauma requires careful attention to patient selection and surgical techniques. Many of these considerations are similar to those for any trauma laparoscopy; however, some are uniquely magnified in a single-port setting.

### 6.1. Patient Selection

Successful application of SPLS in trauma hinges on two overarching prerequisites: the patient must be hemodynamically stable, and the injury pattern must be localized to a limited anatomic region where contamination or hemorrhage can be controlled expeditiously. As with conventional laparoscopy, stability is paramount; patients in shock or with peritonitis from massive hemorrhage require immediate open surgery or at least a multi-port approach with damage control [14,37]. Within this context, current evidence and accumulated institutional experience allow the injuries to be stratified into three practical categories. First, certain injuries are clearly amenable to a single-incision approach. Typical examples include an isolated hollow-viscus perforation, such as a solitary abdominal stab wound with minimal hemoperitoneum, isolated diaphragmatic lacerations, contained serosal injuries of the stomach or duodenum, and selected intraperitoneal bladder injuries (e.g., isolated dome lacerations) that can be repaired with a two-layer closure [38]. In each instance, the pathology can be visualized and definitively managed through umbilical access without the morbidity associated with additional trocars, while maintaining terse operative times and low conversion rates. A second group of lesions is potentially amenable, provided that case selection is meticulous. Minor solid organ trauma limited to solid organ capsular injuries, mesenteric hematomas without arterial extravasation, and isolated omental or epiploic bleeding may all be addressed through SPLS [38]. If more severe injuries are found, significant bleeding occurs, or the operative time becomes excessively prolonged, the surgeon can promptly modify the approach by inserting additional ports (upgrading to standard multi-port laparoscopy), extending the single incision into a mini-laparotomy (for exteriorization or hand assistance), or converting to a full laparotomy [7,39]. Finally, there remains a subset of injuries that are poorly suited to the single-incision platform. Massive hemoperitoneum exceeding one liter, actively bleeding multi-quadrant solid organ trauma, retroperitoneal hematomas with suspected vascular compromise, combined abdominal injuries accompanied by unstable pelvic fractures, and complex pancreaticoduodenal or biliary disruptions all require rapid exposure, multi-quadrant access, and the ability to perform damage control maneuvers or intricate reconstruction. In these circumstances, attempting SPLS risks delaying definitive hemorrhage or contamination control and may jeopardize patient outcomes; multi-port laparoscopy or conventional laparotomy remains the preferred strategy [39].

### 6.2. Important Selection Criteria and Technical Factors for SPLS in Abdominal Trauma

#### 6.2.1. Location of Injury Suspicion

Single-port access through the umbilicus provides a central vantage point; however, extreme upper or lower abdominal areas can be further away from the scope [40]. If an injury is suspected in the pelvis or high in the sub-diaphragmatic area, surgeons must ensure adequate visualization [41]. Patient positioning (Trendelenburg for the pelvis and reverse Trendelenburg for the sub-diaphragm) is critical to facilitate this [13]. In patients with obesity or those with high-riding diaphragms, it may be challenging to see the area of interest from an umbilical port, in which case additional ports may be required for retraction or a better view angle.

#### 6.2.2. Surgeon Experience and Skills

SPLS is associated with a significant learning curve. Maneuvering multiple instruments through a single portal leads to “sword-fighting” (instrument clashing) and loss of the traditional triangulation that surgeons rely on in laparoscopy [10]. Therefore, SPLS for trauma should be performed by surgeons who are comfortable with advanced laparoscopy. Current evidence suggests that experienced laparoscopic surgeons can safely master the single-port technique and that, with practice, the challenges can be overcome [27]. In trauma, where time is essential, the surgeon must be proficient in performing SPLS to avoid undue delays. During the early learning curve, it may be wise to have a low threshold for inserting an extra port if the instrumentation becomes cumbersome [42,43]. Moreover, team familiarity with the technique helped when assistants and scrub nurses knew how to manage single-port instruments [44]. No formal case number threshold has been established for a surgeon to adopt SPLS. In practice, a surgeon experienced in multi-port laparoscopic procedures (e.g., able to perform a laparoscopic cholecystectomy) can generally transition to SPLS with appropriate training. For example, at our institution, any fully trained attending trauma surgeon with adequate laparoscopic experience is permitted to perform SPLS, and we do not require a specific number of prior laparoscopic cases since all trauma surgeons are board-certified specialists. We also do not distinguish between blunt and penetrating trauma in terms of who may perform SPLS, as the technique is only applied in hemodynamically stable patients (usually with preoperative CT imaging). However, penetrating injuries often demand more extensive retroperitoneal exploration than blunt injuries, which can be more technically challenging; accordingly, a higher level of laparoscopic proficiency is advantageous for SPLS in those scenarios.

#### 6.2.3. Equipment and Instrumentation

Having the correct tools can greatly facilitate performing single-port surgery. Ideally, the surgeon should use a purpose-built single-port device that accommodates a laparoscope and two to three instruments through a single incision with gas insufflation [45]. If commercial ports are not available, a single makeshift port (e.g., wound protector and glove technique) can be used. A 30- or 45-degree angled laparoscope often improves the view by allowing for “looking around corners” within the abdomen. Flexible-tip laparoscopes are useful because they can be positioned within a cavity [46]. Articulating or pre-bent instruments for grasping and suturing have been specifically developed to ease single-port surgery by enabling triangulation internally [13]. These can be extremely helpful when attempting therapeutic maneuvers, such as suturing a laceration through a single port. However, many surgeons have reported that standard straight laparoscopic instruments can be used effectively in SPLS with careful technique [13]. One trick is to alternate the instrument hands at different depths (one instrument working closer to the tip of the scope and another working further away) to reduce clashing. The most commonly encountered difficulty in single-port laparoscopic surgery is so-called “sword-fighting,” in which laparoscopic instruments interfere with each other and complicate the procedure [47]. Frequently rotating and angling instruments are deemed necessary. Another useful technique is a cross-handed operation, in which the surgeon’s left hand controls an instrument that emerges on the right side internally and vice versa, which restores a semblance of triangulation at the target site [13]. However, these maneuvers require practice. Additionally, ensuring the single-port incision (usually at the umbilicus) is of adequate length (often 2.0–2.5 cm) to allow instrument freedom and prevent crowding at the skin level is important; a small incision can act as a tight fulcrum and create friction on instruments, so enlarging it by a few millimeters can improve mobility.

#### 6.2.4. Adjunct Techniques

In trauma SPLS, adjuncts such as hand assistance or mini-laparotomy extraction should be employed, if needed. Hand-assisted laparoscopy involves inserting a surgeon’s hand through a small incision (often the same umbilical incision, slightly extended) with a hand-port device, which can greatly aid palpation, retraction, and rapid control of bleeding. While this technically breaks the pure single-port approach (since the hand acts as an “assistant”), it can be life saving in a difficult situation and still avoids a full-length laparotomy incision. Similarly, as shown by Yang et al. [31], converting a single incision into a mini-laparotomy to exteriorize the bowel for repair is a useful hybrid strategy. These strategies should be planned in advance; for example, during a single-port exploration, if a significant bowel injury is found to be tedious to sew intracorporeally, the surgeon can enlarge the incision and fix it externally. This flexibility is key to safely adopting SPLS in trauma; rather than being all or nothing, one can start with a single port and escalate as needed. Indeed, the ability to easily add ports is one of the advantages of SPLS in trauma, and converting from one port to multiple laparoscopic ports spares the patient from undergoing open laparotomy [19].

#### 6.2.5. Safety and Bailout

The surgical team must establish clear criteria for deciding when to abandon the single-port approach. The single-incision platform must be abandoned as soon as it compromises either visualization or patient stability. Conversion is warranted when bleeding persists despite routine laparoscopic maneuvers; for example, continued arterial oozing after topical hemostatic agents or suction packing, or when the operative field repeatedly clouds with blood or distended bowel that cannot be cleared. Any trend toward physiological deterioration despite resuscitative efforts, such as a rising heart rate, falling blood pressure, or escalating vasopressor requirements, should likewise prompt escalation. Discovery of injuries that clearly demand complex reconstruction, including complex pancreatic injury, a major mesenteric or iliac vessel injury, or a full-thickness bladder rupture near the trigone, is a further signal to leave the single port in favor of an open midline incision [37]. Persistent instrument interference, difficulty visualizing injuries located far from the single port, or situations that require intricate intracorporeal suturing also favor early transition to a multi-port approach. Patient safety should not be compromised when using a single-incision approach. When properly implemented, conversion is not a failure but rather a judicious decision, and as noted earlier, trauma surgeons commonly report conversion rates in the range of 15–30% for diagnostic/therapeutic laparoscopy [8,14].

### 6.3. Outcomes and Specific Considerations

The reported outcomes of SPLS for trauma have been favorable. No increase in the overall complication rate was observed in small cohorts [29,31]. One concern with a single umbilical incision is the risk of incisional hernia because the fascial defect is larger than the standard 5 mm trocar site. In the elective single-port surgery literature, umbilical hernia rates have been low, but not zero. For instance, one large series of over 100 general surgery SILS cases revealed an umbilical herniation rate of approximately 4% [48]. With proper closure of the fascial layer, most studies have concluded that single-port surgery does not pose a significantly higher risk of herniation than conventional laparoscopy [26,49]. In patients with trauma, especially younger patients, wound healing is usually good; however, this underscores the importance of diligently closing the umbilical fascia—often in a J- or U-shaped manner if a port device is used. Another consideration is pain; intuitively, one might expect less pain with a single incision than with multiple incisions, but the single incision is somewhat larger and can be subject to more torque from the instruments. The evidence on pain is mixed—some studies in other fields have reported a mild reduction in pain with a single port, while others reported no difference [23,27]. However, none of the patients experienced worse pain; therefore, the patients at most experienced discomfort equivalent to that of standard laparoscopy. Cosmesis is clearly improved as the scar is hidden in the umbilical fold. Patients with trauma may have other scars or injuries, but minimizing surgical scars is still beneficial for long-term satisfaction.

In summary, the technical keys to successful SPLS in patients with trauma include selecting stable patients with likely manageable injuries, using appropriate single-port equipment, leveraging advanced laparoscopic skills, such as articulating instruments and optimal positioning, and maintaining a low threshold to adjust the strategy through adding ports or open conversion whenever needed. When these principles are followed, single-port laparoscopy can be integrated into trauma practice with outcomes comparable to multi-port laparoscopy [3,18].

## 7. SPLS Intracorporeal and Extracorporeal Examination and Procedure

### 7.1. Authors’ Method

Patients who are hemodynamically unstable are excluded from SPLS and undergo immediate laparotomy [50]. On the other hand, based on our experience, most patients with hemodynamically stable abdominal trauma can be managed using a single-port laparoscopic approach that combines intracorporeal diagnostic exploration with extracorporeal organ examination and intervention. As these patients have stable vital signs, preoperative imaging (typically an abdominal CT scan) is performed to localize the injuries. This hybrid single-incision approach is especially suited for suspected hollow viscus injuries (e.g., bowel perforations) and scenarios of unclear diagnosis with intraperitoneal fluid on imaging [50]. In such cases, SPLS can confirm or rule out bowel perforation and address it, thus avoiding full laparotomy. Patients with suspected isolated bowel injuries (without liver or splenic injury on CT) are ideal candidates for this method.

### 7.2. Surgical Setup and Access

The patient is placed in a supine position (for standard laparoscopy) with the arms positioned for anesthesia access, and the operating table is prepared to allow tilting as needed during the procedure. The surgeon typically stands at the patient’s left side to facilitate the running of the bowel, and a standard laparoscopic tower is positioned. Access is gained through a single umbilical incision, measuring approximately 3 cm in diameter. An Alexis wound protector (Applied Medical Resources Corp., Rancho Santa Margarita, CA, USA) is inserted into the transumbilical incision, and a sterile surgical glove is secured over the protector’s outer ring to create a homemade multichannel glove port. Through small openings in the fingers of the glove, multiple trocars (usually one 10–12 mm and two 5 mm) are placed for the laparoscope and instruments, and a pneumoperitoneum is established (typically to a pressure of approx. 12 mmHg). This cost-effective single-port device provides access to a 0° or 30° laparoscope and standard or articulating laparoscopic instruments; the specific configuration can be adjusted as needed. The use of the umbilical site renders the incision essentially hidden and easily extendable, if necessary. Once the glove port is secured and insufflation achieved, a thorough laparoscopic exploration is performed.

### 7.3. Intracorporeal Exploration Phase

**Figure 1:**
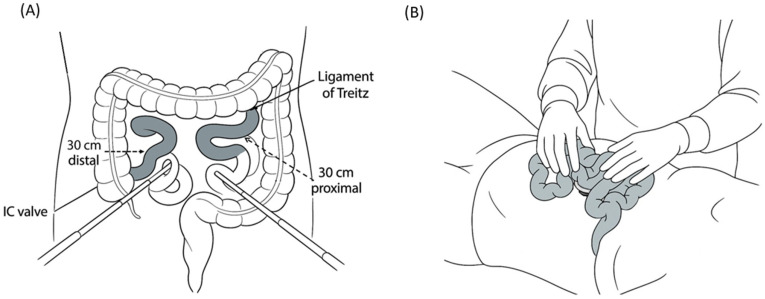
Intracorporeal and extracorporeal inspection of the small intestine. Intracorporeal inspection is first performed for the segments within 30 cm distal to the ligament of Treitz and 30 cm proximal to the ileocecal valve (**A**), which cannot be exteriorized. The remaining mid-portion is then inspected extracorporeally for convenience (**B**).

Using single-port access, the surgeon first conducts a systematic diagnostic laparoscopy of the abdomen. Any active intra-abdominal bleeding is immediately addressed, typically by laparoscopic hemostasis using electrocautery or direct pressure, with priority given to hemorrhage control. Blood and clots are suctioned out to clear the field, and the extent of any contamination, such as intestinal content spillage, is assessed. The surgeon then inspects the regions that are often missed on imaging, specifically the undersurface of the diaphragm and bladder dome, to identify occult injuries that preoperative CT may not have definitively shown (small diaphragmatic tears or bladder injuries can be missed on scans). Thorough inspection of the solid organs, focusing on the liver and spleen, is performed. If the CT scan did not indicate injuries to the liver or spleen, and no injuries are observed laparoscopically, the surgeon concentrates on the evaluation of the gastrointestinal tract. The entire colon is examined laparoscopically in sequence (visualizing the cecum; ascending, transverse, and descending colon; and rectosigmoid to the furthest extent) to check for serosal tears, hematomas, or perforations. Thereafter, the small intestine is examined, where the authors’ technique diverges from a purely intracorporeal approach. Running the entire small bowel with a laparoscope is difficult and time consuming and risks missing segments or causing iatrogenic injury while handling instruments [51]. Therefore, the surgeon limits laparoscopic small bowel examination to the proximal and distal segments that are difficult to exteriorize rather than attempting to extensively inspect the whole bowel internally. Specifically, the first 30 cm of the small bowel distal to the ligament of Treitz and the last 30 cm proximal to the ileocecal valve are inspected intracorporeally (Figure 1A). The duodenojejunal and distal ileum are laparoscopically visualized for injury because the proximal and distal small bowel loops are relatively fixed and not easily exteriorized. If bowel perforation or mesenteric injury is identified in these segments, intracorporeal repair can be attempted using laparoscopic suturing or stapling. In practice, such injuries in these segments are uncommon [52], and the initial laparoscopic inspection of these areas is typically negative for major injuries. The limited laparoscopic “run” of the bowel greatly saves time compared to examining the entire small intestine laparoscopically. After completing the focused intra-abdominal examination, any free fluid or contamination is irrigated and suctioned laparoscopically to the furthest possible extent. At this point, the surgeon has inspected all key areas, including the diaphragm, bladder, solid organs, colon, and proximal and distal small bowel loops. Most sections of the small intestine are extracorporeally evaluated in the next phase (Figure 1B).

### 7.4. Extracorporeal Evaluation and Intervention Phase

**Figure 2:**
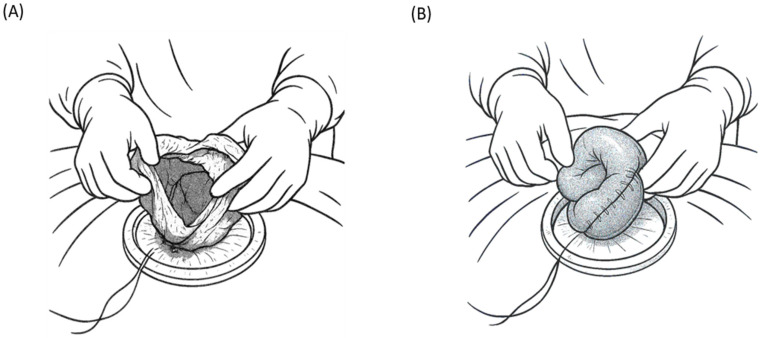
Extracorporeal management of the small intestine. The injured small bowel segment is exteriorized (**A**) and managed extracorporeally, allowing resection and anastomosis (**B**) in a manner similar to open surgery.

After the transition in the procedure to mini-laparotomy using the same umbilical incision, the pneumoperitoneum is released and the glove portion of the single-port device is removed, leaving the Alexis wound retractor in place to protect the incision. This effectively converts access into an open surgical window (approximately 3 cm, extendable if needed) at the umbilicus, through which the surgeon can directly insert fingers or deliver abdominal content. Through this incision, the surgeon sequentially exteriorizes the remaining small bowel—the mid-jejunum through the mid-ileum, which is not fully examined laparoscopically. Small bowel loops are gently removed through the umbilical wound and inspected manually and visually in the operative field. The surgeon “runs” the bowel extracorporeally, palpating and inspecting the antimesenteric and mesenteric borders for any injuries. If an injury area is observed, such as a small perforation, serosal tear, or mesenteric bleeding, it can be addressed immediately and safely outside the abdomen (Figure 2A). For example, small perforations or lacerations can be sutured directly under vision, and bleeding points can be ligated or cauterized. If a segment of the bowel is devascularized or severely damaged such that primary repair is not feasible, segmental resection and anastomosis can be performed extracorporeally (Figure 2B). Conducting these repairs in the open field through a mini-incision is typically faster and technically easier than intracorporeal laparoscopic suturing, particularly in cases of urgent trauma. This hybrid approach takes advantage of the single incision of the laparoscopic port and mini-laparotomy site, allowing rapid conversion to external manual handling without the need for a formal large laparotomy [31,53]. Once the entire small intestine has been examined and any injuries are treated, the bowel is returned to the abdominal cavity. The abdomen is thoroughly irrigated with warm saline to remove blood and contaminants. Irrigation is performed through an umbilical incision (a mini-laparotomy), often by pouring saline into the abdomen with a bulb syringe while repositioning the operating table (Trendelenburg/reverse Trendelenburg or lateral tilt) to direct the fluid into all recesses. A large-bore suction device is used to aspirate fluid and debris to ensure that the peritoneal cavity is clean. After irrigation and suction, a drain may be placed if indicated. Notably, by employing this technique, the drain is inserted through an existing umbilical incision/port site, eliminating the need for creating additional incisions for drain placement. The drain (typically a soft Jackson–Pratt drain) is guided through the umbilical opening and positioned with its tip in the pelvic cavity (the most dependent area) to evacuate the residual fluid. Finally, the umbilical wound is closed in layers. The fascial defect (approximately 3 cm or slightly larger if extended) is securely sutured to prevent hernia, and the skin at the umbilicus is reapproximated, often resulting in a scar hidden within the umbilical fold.

### 7.5. Special Considerations

In most cases, managed using the SPLS hybrid technique, primary injuries involve the small intestine, and the aforementioned steps allow for the efficient and safe completion of the operation. There are a few exceptional scenarios that merit adjustments to this approach. First, if a bladder injury is present (e.g., bladder dome laceration from trauma), it is preferred to be repaired laparoscopically during the intracorporeal phase. Bladder dome injuries in blunt trauma are often accessible and visible laparoscopically, and a two-layer suture repair can be performed entirely under SPLS without needing an extracorporeal step (a postoperative Foley catheter is placed) [30,54]. Second, in the case of a diaphragmatic injury, reaching the injury from an umbilical single port alone can be challenging owing to the distance and angle (especially for high posterolateral diaphragm lacerations) [55]. If the diaphragmatic tear is not easily accessible with instruments through the umbilical port, additional laparoscopic ports are inserted (e.g., in the subcostal area) to grant the proper angle for suturing the diaphragm. This technique avoids the need to conduct a full laparotomy, converting the procedure to multi-port laparoscopy as needed to achieve secure diaphragmatic repair [55]. These two scenarios (bladder and diaphragmatic repairs) were relatively uncommon in our series of SPLS cases, as patients with such injuries often have other injuries and may require tailored approaches. The third scenario, which was more frequently encountered, is trauma to the sigmoid colon. Mobility (redundancy) of the sigmoid colon varies among individuals. In some cases, similar to the small bowel, the sigmoid colon can be easily delivered through an umbilical incision for extracorporeal inspection and repair. However, if the sigmoid colon is injured (e.g., a perforation) and is not sufficiently mobile for externalization, the surgeon can perform laparoscopic mobilization of the sigmoid (dividing some supporting ligaments or lateral peritoneal attachments) to free it [56,57]. Once mobilized, the injured sigmoid segment is pulled out through the umbilical incision, and the necessary procedure (such as primary repair or resection with anastomosis) is performed externally. In such a case, it may be necessary to enlarge the umbilical incision by an extra 2–3 cm to accommodate the exteriorized colon and facilitate repair. Even with this extension, the incision remained much smaller than that in a conventional midline laparotomy. These adaptations demonstrate the flexibility of the single-incision approach; additional ports or a slight extension of the incision can be selectively employed to ensure that all injuries are addressed without abandoning the minimally invasive strategy, unless absolutely necessary. Importantly, the threshold for conversion to open surgery remains low if needed for patient safety; however, in practice, with proper patient selection, conversion is rarely required.

### 7.6. Advantages of the Hybrid Single-Port Approach

This intracorporeal-plus-extracorporeal SPLS technique offers several advantages over traditional multi-port laparoscopy and open laparotomy. By leveraging laparoscopy for the initial survey, the approach retains all the well-known benefits of minimally invasive surgery: a smaller incision (only one small umbilical wound), reduced risk of wound complications, such as infection and hernia, less postoperative pain, faster recovery, and shorter hospital stays [3,14]. Moreover, by incorporating an extracorporeal phase for small bowel evaluation, the surgeon can safely and confidently inspect the entire intestine with direct palpation and visualization, which could not be efficiently accomplished by pure laparoscopy. This reduces the likelihood of missed subtle injuries (e.g., tiny perforations or mesenteric tears) and avoids the need to run the bowel intracorporeally for prolonged periods [14]. In principle, the method combines the strengths of laparoscopy and open surgery; it achieves a thorough exploration and repair similar to those of an open operation while confining the intervention to a single small incision. Time efficiency is another significant benefit, where, in our practice, the total operative time of this hybrid technique was comparable to that of formal laparotomy; the time spent in the limited laparoscopic examination was offset by the rapid extracorporeal treatment of injuries and quicker wound closure (a small single incision is faster to open and close than a long midline incision). By avoiding multiple trocar insertions and extended intracorporeal suturing, the procedure minimizes the “sword-fighting” of instruments and fatigue associated with long, complex laparoscopy, potentially shortening the operative duration. Additionally, if an unexpected finding requires wider exposure, the existing umbilical incision can be swiftly extended to convert it into a formal open laparotomy, providing a safety net without requiring new incisions. Lastly, our method has a relatively short learning curve—by minimizing the complex, purely intracorporeal components, it can be easily adopted, even by surgeons with moderate laparoscopic experience.

Overall, this hybrid single-port approach is feasible and safe and effectively balances invasiveness with thoroughness. This allows trauma surgeons to capitalize on the benefits of minimally invasive surgery without compromising the completeness of injury identification or the quality of repair. Early case reports and our institutional experience support that single-incision laparoscopy with selective extracorporeal techniques can definitively manage bowel injuries while avoiding the risk of morbidity associated with a full laparotomy [31,53]. In summary, compared with traditional approaches, the SPLS intracorporeal/extracorporeal technique appears to offer the best for patients with trauma, ensuring a thorough small bowel evaluation, maintaining a short operative time, and significantly reducing wound size and associated complications. This real-world approach further underscores the potential of single-port laparoscopy as a powerful tool for trauma surgery, bridging the gap between conventional multi-port laparoscopy and open surgery.

## 8. Future Directions

SPLS for trauma is an evolving field, and several developments on this horizon promise to expand its role and efficacy. Given the currently limited but encouraging evidence, future efforts will likely focus on refining the technique, improving the technology, and accumulating more robust clinical data.

### 8.1. Increasing Adoption and Training

With the increase in the number of trauma surgeons who gain experience in advanced laparoscopy, SPLS is expected to be adopted in additional trauma centers. The learning curve is aided by dedicated training in single-port techniques, including simulation-based practices [58]. Workshops and courses on SILS have become more common in general surgery, and similar educational opportunities specific to trauma scenarios, such as managing trauma models through a single port, may emerge. With the increase in the technique’s familiarity over time, the indications for its use can be broadened [59]. For example, surgeons may begin to routinely use single-port laparoscopy for the diagnostic evaluation of patients with stable blunt trauma or for specific injuries, such as isolated diaphragmatic tears. Widespread adoption depends on institutional support, including the availability of single-port devices. If hospitals invest in single-port equipment as a standard OR asset, surgeons are more likely to incorporate this technique in urgent cases. Notably, as of 2023, international trauma guidelines recognized the importance of minimally invasive approaches, and the World Society of Emergency Surgery consensus recently suggested a laparoscopic-first approach for stable patients requiring emergency abdominal surgery, including trauma cases [60]. This endorsement of laparoscopy in general will likely encourage the exploration of the single-port variant.

### 8.2. Clinical Research and Evidence Building

More data on SPLS for trauma are warranted. Future research should include multicenter case series or registries that track the outcomes of trauma SPLS to better define its safety and efficacy. While randomized controlled trials in trauma are challenging—given the acute decision-making and diverse injury patterns—prospective observational studies could compare cohorts managed with single-port and multi-port laparoscopy. The important outcomes to be assessed are diagnostic accuracy (missed injury rate), procedure time, conversion rate, complication rate, pain levels, cosmetic satisfaction, and overall length of hospital stay. Currently, the literature is too sparse to determine whether SPLS offers a statistically significant improvement in outcomes, such as pain or recovery from trauma. The main benefit could solely be cosmetic, or there may be subtle advantages with respect to reducing the risk of morbidity that larger studies can reveal. Conversely, research may identify specific scenarios in which a single port is not beneficial, and high-quality data are needed to guide the best practices. The current consensus, based on low-level evidence, is that SPLS is not inferior to traditional laparoscopy for carefully selected cases [18,29]. Demonstrating this on a larger scale is important for surgeons’ confidence and guideline inclusion.

### 8.3. Technological Advancements

The future of single-port surgery is closely tied to technological innovation. One of the most promising areas of research is robotic single-port surgery. Robot-assisted laparoscopic systems (such as the da Vinci platform) offer enhanced dexterity, wristed instrumentation, and 3D visualization, which can overcome the ergonomic challenges of single-incision surgery [61]. In fact, robots can eliminate most instrument collisions using internal articulation and restore true triangulation even through one port [61]. A dedicated single-port robotic platform (da Vinci SP) was introduced, allowing three or four robotic instruments and a camera to be deployed through a single 2.5 cm cannula [61]. Early experiences with single-port robotics in urology and colorectal surgery show that it achieves comparable outcomes to multi-port surgery with the potential for less surgeon strain [61,62,63]. Evidence for robotic surgery, particularly robotic single-port surgery in trauma patients, is extremely limited; only a handful of reports describe its use in emergencies, such as hemorrhagic colon cancer or perforated peptic ulcers [64,65]. Given the expanding role of robotics in other emergent operations, however, it is plausible that carefully selected trauma patients could also benefit from this technology [66]. For example, one could imagine a scenario in the future in which a trauma surgeon in a hybrid emergency room/OR uses a single-port robot to perform rapid diagnostic exploration and emergency interventions, such as suturing a liver laceration, with improved precision. Nonetheless, the substantial financial burden of robotic systems, the need for specialized instruments, and the requirement for a highly trained team remain significant obstacles in urgent settings. Robust clinical data are, therefore, needed to establish both the efficacy and safety of robotic approaches before they can be widely adopted in acute trauma care.

### 8.4. Protocol Integration and Hybrid Approaches

Over the upcoming period, trauma algorithms may formally integrate single-port laparoscopy. For instance, protocols can be developed for single-port diagnostic laparoscopy in stable blunt trauma after equivocal imaging, single-port peritoneal lavage, and assessment of penetrating injuries. Hybrid trauma management, which combines endovascular, laparoscopic, and open techniques, is an active research area [67,68]. SPLS could play a role in hybrid damage control surgery; for example, after endovascular embolization of a splenic injury, single-port laparoscopy might be used to inspect the abdominal cavity for other injuries or to confirm bleeding control [69]. The flexibility to extend the single incision, if needed, implies that SPLS can be a component of staged approaches (diagnostic laparoscopy first, followed by mini-laparotomy if required) with minimal additional invasiveness. As trauma systems continue to emphasize reducing non-therapeutic operations, minimally invasive diagnostics, such as SPLS, will gain attention.

### 8.5. Long-Term Outcomes and Quality of Life

Future studies should explore beyond immediate surgical outcomes to long-term patient-centered outcomes. Patients with trauma who avoid large laparotomies may have fewer challenges with ventral hernias and bowel adhesions. They may also experience better functional recovery and an earlier return to work. Quantifying these benefits will help justify the use of these minimally invasive methods. Quality-of-life assessments comparing scars and pain between single-port, multi-port, and open surgeries could highlight the value of SPLS. Particularly for young patients or those concerned about body image, the near-invisible scarring of a single-port procedure can positively affect psychological recovery.

## 9. Conclusions

SPLS is at the forefront of innovation in minimally invasive trauma care. The current trajectory suggests that it will become an increasingly common tool employed by trauma surgeons as evidence and experience accumulate. A recent expert consensus has advocated a laparoscopic-first approach for stable abdominal trauma management [60]; SPLS will likely be one of the modalities through which this vision is realized. Continued advancements in technology, along with training and research, will address these remaining challenges. SPLS may transition from an “emerging alternative” to a standard approach for managing abdominal trauma, offering patients the dual benefits of effective injury management and the least invasive surgery.

## Abbreviations

The following abbreviations are used in this manuscript:

CT	Computed tomography
NOTES	Natural orifice transluminal endoscopic surgery
SILS	Single-incision laparoscopic surgery
SPLS	Single-port laparoscopic surgery
